# Mitochondrial transplantation attenuates hypoxic pulmonary vasoconstriction

**DOI:** 10.18632/oncotarget.8893

**Published:** 2016-04-21

**Authors:** Juan Zhou, Jiwei Zhang, Yankai Lu, Songling Huang, Rui Xiao, Xianqin Zeng, Xiuyun Zhang, Jiansha Li, Tao Wang, Tongfei Li, Liping Zhu, Qinghua Hu

**Affiliations:** ^1^ Department of Pathophysiology, School of Basic Medicine, Tongji Medical College, Huazhong University of Science and Technology, Wuhan 430030, China; ^2^ Key Laboratory of Pulmonary Diseases of Ministry of Health, Tongji Medical College, Huazhong University of Science and Technology, Wuhan 430030, China; ^3^ Department of Pathology, Union Hospital, Tongji Medical College, Huazhong University of Science and Technology, Wuhan 430022, China; ^4^ Department of Pathology, Tongji Hospital, Tongji Medical College, Huazhong University of Science and Technology, Wuhan 430030, China; ^5^ Department of Respiratory and Critical Care Medicine, Tongji Hospital, Tongji Medical College, Huazhong University of Science and Technology, Wuhan 430030, China; ^6^ Department of Pathology, School of Basic Medical Sciences, Hubei University of Medicine, Shiyan 442000, China; ^7^ Current address: Department of Clinical Laboratory of Xuzhou Central Hospital, Xuzhou 221009, China

**Keywords:** hypoxia, reactive oxygen species, calcium signaling, pulmonary vasoconstriction, mitochondria

## Abstract

Hypoxia triggers pulmonary vasoconstriction, however induces relaxation of systemic arteries such as femoral arteries. Mitochondria are functionally and structurally heterogeneous between different cell types. The aim of this study was to reveal whether mitochondrial heterogeneity controls the distinct responses of pulmonary versus systemic artery smooth muscle cells to hypoxia. Intact mitochondria were transplanted into Sprague-Dawley rat pulmonary artery smooth muscle cells in culture and pulmonary arteries *in vitro*. Mitochondria retained functional after transplantation. The cross transplantation of mitochondria between pulmonary and femoral artery smooth muscle cells reversed acute hypoxia-induced alterations in cell membrane potential, [Ca^2+^]_i_ signaling in smooth muscle cells and constriction or relaxation of arteries. Furthermore, the high or low amount of reactive oxygen species generation from mitochondria and their divergent (dis-)abilities in activating extracellular Ca^2+^-sensing receptor in smooth muscle cells were found to cause cell membrane potential depolarization, [Ca^2+^]_i_ elevation and constriction of pulmonary arteries versus cell membrane potential hyperpolarization, [Ca^2+^]_i_ decline and relaxation of femoral arteries in response to hypoxia, respectively. Our findings suggest that mitochondria necessarily determine the behaviors of vascular smooth muscle cells in response to hypoxia.

## INTRODUCTION

Mitochondria are critical in the initiation of hypoxia-induced pulmonary vasoconstriction (HPV) [1, 2, 3, 4, 5]. HPV maintains physiological ratio of lung respiration and blood perfusion and contributes to the pathophysiologic development of pulmonary hypertension and pulmonary edema. By contrast, hypoxia triggers relaxation of systemic vessels [2, 6, 7]. Mitochondria in pulmonary artery smooth muscle cells (PASMCs) appear structurally and functionally distinct from systemic artery SMCs [1, 2]. Whether the difference between mitochondria represents the mechanism underlying distinct responses to hypoxia between pulmonary and systemic arteries is undetermined according to Koch's Postulates [2, 5, 8]. It warrants investigation employing a novel experimental strategy to illustrate the cause-effect relationship between mitochondria and the responses of SMCs to hypoxia. Additionally, mitochondria are active organelles [9, 10], whose function depends on their fusion, fission and/or division under (patho-)physiological circumstances such as hypoxia [10]. Logically, the replacement and/or mixture of different mitochondria can be expected to change their behaviors [9].

Here we showed that isolated mitochondria can be delivered or transplanted into SMCs in culture and SMCs of pulmonary arteries (PAs) *in vitro*. We found that the transplantation of mitochondria derived from femoral artery smooth muscle cells (FASMCs) inhibited hypoxia-induced cell membrane potential depolarization and [Ca^2+^]_i_ elevations in cultured PASMC preparations, attenuated hypoxia-induced constriction of isolated PAs *in vitro*, and *vice versa*. We also identified the mechanisms underlying the effects of transplanted mitochondria, which was associated with the potent capability of generating reactive oxygen species (ROS) in PASMCs versus FASMCs in response to hypoxia. Our study points to the determinant role of mitochondria in vascular SMC responses to hypoxia.

## RESULTS

### Transplantation of mitochondria into vascular SMCs in culture

**Figure 1:**
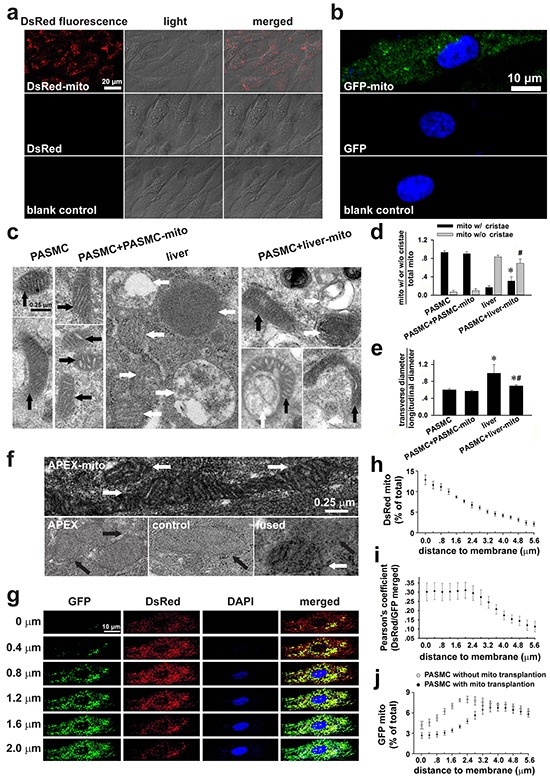
Transplantation of exogenous mitochondria into PASMCs in culture **a.** Live cell confocal imagings of PASMCs after incubation with DsRed-labeled mitochondria (prepared from PASMC, upper), DsRed (middle) or vehicle (lower, n=3). **b.** Indirect immunofluorescent-stainings of the fixed PASMCs after incubation with GFP-labeled mitochondria (prepared from PASMC, upper and lower), GFP (middle) with (upper and middle) or without antibody against GFP (lower, n=3). **c-e.** Electron micrographs (EM) showing long spindle mitochondria with clear cristae in control PASMCs and PASMCs incubated with PASMC-derived mitochondria (PASMC-mito) (black arrows, left, **c**), round mitochondria with swelling, unclear cristae in Wilson's rat liver cells (white arrows, middle, **c**), mixture of mitochondria with distinct morphology in PASMCs after incubation with the liver mitochondria (liver-mito) (black and white arrows, right, **c**) and quantitative comparisons of two shapes of mitochondria (* # *p* < 0.05 *vs.* mito with/without cristae in PASMCs respectively, **d**). as well as the ratio of width to length of mitochondria (*p < 0.05 *vs.* PASMCs, #*p* < 0.05 *vs.* liver, **e**). Quantitation obtained from 58, 124, 232 and 73 mitochondria of 12, 11, 20 and 11 cells for control PASMCs, PASMCs incubated with PASMC-mito, liver cells and PASMCs incubated with liver-mito, respectively. **f.** The representative images showing apparent EM contrast only in the mitochondrial matrix, not the intermembrane space in PASMCs after transplantation of APEX-labeled mitochondria (white arrows, upper), no EM contrast in mitochondria in PASMCs with incubation of APEX (black arrows, left, lower) or in control PASMCs without transplantation of APEX-labeled mitochondria (black arrows, middle, lower), and the mixture/fusion of exogenous mitochondria with EM contrast and endogenous counterpart without EM contrast in the PASMCs after transplantation of APEX-labeled mitochondria (white and black arrows, right, lower) (n=3 for each). **g-j.** Continuous confocal scannings of DsRed-labeled mitochondria transplanted into PASMC with GFP-labeled endogenous mitochondria (**g**, the distance away from cell surface shown on the left of each panel), quantitative distributions of DsRed-mitochondria by the distance to cell membrane (**h**), Pearson's coefficient showing co-localizations of DsRed- and GFP-mitochondria by the distance to cell membrane (**i**) and distribution of GFP-labeled endogenous mitochondria by the distance to cell membrane in PASMCs with transplantation of DsRed-labeled exogenous mitochondria versus those in PASMCs without transplantation of exogenous mitochondria (**j**) (n=6 for **g-j**).

To determine whether exogenous mitochondria can be transplanted into SMCs, PASMCs in culture were incubated with DsRed-labeled mitochondria. After 24 hours incubation and washout of medium containing DsRed-labeled mitochondria, DsRed fluorescence was identified by live cell confocal imaging to locate within the PASMCs (Figure 1a, upper), not in the control cells (Figure 1a, lower). The successful transplantation of exogenous mitochondria was verified by immunocytofluorescent stainings of the fixed PASMCs after incubation with GFP-labeled mitochondria (upper, Figure 1b). The incubation of PASMCs with DsRed or GFP protein at the concentration of 0.76 μg/ml, yielding fluorescent intensity equal to the DsRed or GFP-labeled mitochondria used, did not result in any internalization of DsRed or GFP within PASMCs (Figure 1a, middle and Figure 1b, middle), indicating that DsRed or GFP-labeled mitochondria transplanted into PASMCs were intact organelles rather than merely endocytosed DsRed protein aggregates.

Furthermore, PASMCs were incubated with mitochondria prepared from Wilson's disease rat liver [11], which were round with swelling, unclear or disappeared cristae (Figure 1c, middle). These features were distinct from the long spindle mitochondria with clear cristae in PASMCs (Figure 1c, left). In PASMCs incubated with the liver mitochondria, mitochondria were diverse in shape including oval and spindle ones with clear cristae as well as round ones with unclear, swelling cristae or without cristae (Figure 1c, right), indicating that the liver mitochondria were transplanted into PASMCs. Some long mitochondria with clear cristae appeared fused with round one (s) without clear cristae (Figure 1c, right). In the parallel control PASMCs, mitochondria in PASMCs incubated with PASMC-derived mitochondria were long with clear cristae, not distinct from those in native PASMCs (Figure 1c, left). Quantitative estimations of the mitochondria with or without clear cristae as well as the ratio of width to length of mitochondria among the four types of cells (Figure 1d–1e) also confirmed the intracellular transplantation of liver mitochondria and possible fusion of exogenous mitochondria with endogenous ones.

To further verify the intracellular delivery of exogenous mitochondria at the ultra-structural level, FASMCs were transfected with mitochondria-targeted vector expressing ascorbate peroxidase (APEX), a genetic label for electron microscopy (EM) [12, 13]. EM examinations showed apparent EM contrast only in the mitochondrial matrix, not the intermembrane space in 56 out of total 170 mitochondria in 29 PASMCs after incubation with APEX-labeled mitochondria (Figure 1f, white arrows, upper), no EM contrast in any of total 67 mitochondria in 13 PASMCs with incubation of 0.76 μg/ml APEX (black arrows, left, lower) or any of total 51 mitochondria in control PASMCs without incubation of APEX-labeled mitochondria (Figure 1f, black arrows, middle, lower), and the mixture/fusion of exogenous mitochondria with EM contrast and endogenous counterpart without EM contrast in the PASMCs after incubation of APEX-labeled mitochondria (Figure 1f, white and black arrows, right, lower).

**Figure 2:**
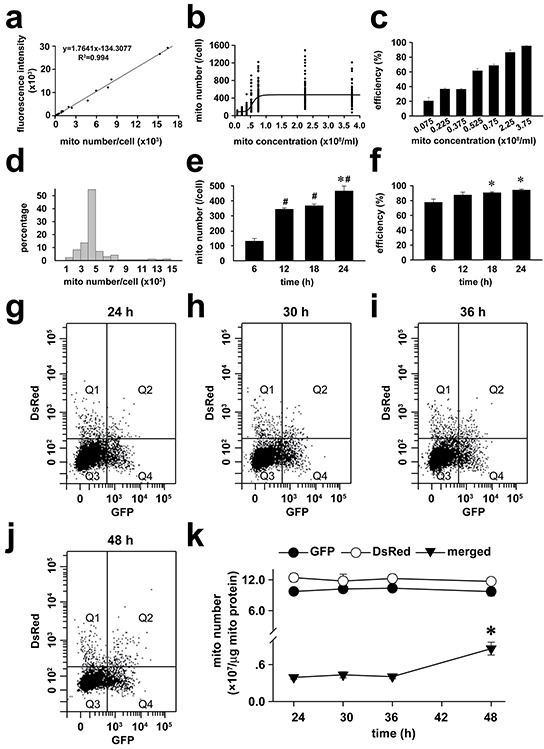
Dynamics of transplantation of exogenous mitochondria into PASMCs in culture **a-f.** Calibration curve between the numbers of isolated mitochondria (mito) and GFP fluorescent intensities (**a**), dependence of intracellular GFP-mitochondrial quantity (**b**) and efficiency of GFP-mitochondria-transplanted cells (**c**) on exogenous mitochondrial concentrations after 24-hour incubation, relative distributions of intracellular GFP-mitochondrial quantity after 24-hour incubation of 2.25×10^8^/ml mitochondria (**d**), time-dependence of intracellular GFP-mitochondrial quantity (# *p* < 0.05 *vs.* 6 h, *p < 0.05 *vs.* 12 or 18 h, **e**) and efficiency of GFP-mitochondria-transplanted cells (* *p* < 0.05 *vs.* 6 h, **f**) after incubating with 2.25×10^8^/ml mitochondria (n=3 for **a-f**). **g-k.** The representative flow cytometry for the separation of DsRed-labeled exogenous mitochondria (Q1), GFP-labeled endogenous mitochondria (Q4) as well as GFP and DsRed-merged mitochondria (Q2) from PASMCs at (**g**) and after (**h**, **i**, and **j**) the accomplishment of a 24 hours incubation with exogenous mitochondria, respectively and statistical summary of the change of mitochondrial number over time (**k**). * *p* < 0.05 *vs* 24 hour, n=3 for each.

To determine the spatial distributions of exogenous mitochondria within PASMCs, the endogenous mitochondria of PASMCs were pre-labeled with GFP and then the PASMCs were incubated with DsRed-labeled exogenous mitochondria. The continuous line scannings of the PASMCs by confocal microscopy revealed predominant localization of DsRed mitochondria in the areas close to the superficial cytoplasma membrane (Figure 1g–1i) and the similar distributions of fused/mixed mitochondria, the merged GFP and DsRed area within the cell (Figure 1g and 1i). The amount of GFP-labeled, endogenous mitochondria in the areas close to the superficial cytoplasma membrane in PASMCs with transplantation of exogenous mitochondria became fewer than those without transplantation (Figure 1j). The confocal line scannings were also assembled to further illustrate the three-dimensional intracellular distributions of transplanted mitochondria in PASMCs (Supplementary Figure S1). The transplantation of exogenous mitochondria into PASMCs clearly exhibited a dynamical dependence on mitochondrial concentrations in the medium (Figure 2b–2c) and the incubation time period (Figure 2e–2f). After transplantation, the number of exogenous mitochondria remained relatively constant for up to 24 hours as determined by GFP and DsRed fluorescence sorting on flow cytomerty (Figure 2g–2k). A small portion of GFP and DsRed-merged mitochondria, probably the fused ones, increased at 24 hours after the accomplishment of transplantation (Figure 2g–2k).

To address the mechanisms underlying how mitochondria can be internalized by the recipient cells, we explored several pathways or factors involved in pinocytosis. Chlorpromazine, the inhibitor of clathrin did not suppress the internalization of DsRed-labeled exogenous mitochondria (Supplementary Figure S2). By contrast, the inhibitor of sodium–hydrogen exchanger, ethylisopropylamiloride (EIPA); the inhibitor of microtubule polymerization, nocodazole; and the inhibitor of actin polymerization, cytochalasin D, significantly inhibited the internalization of DsRed-labeled exogenous mitochondria, respectively (Supplementary Figure S2). The above results suggest that the internalization of exogenous mitochondria in PASMCs involves macropinocytosis, but does not depend on clathrin.

### Mitochondrial transplantation on acute hypoxia-altered [Ca^2+^]_i_ and cell membrane potential

**Figure 3:**
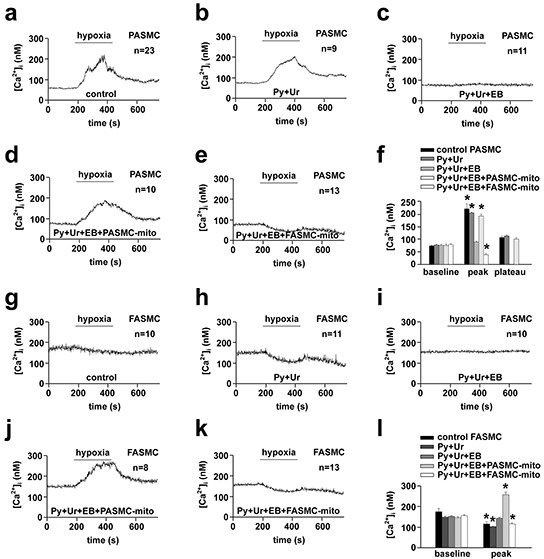
Changes of hypoxia-altered [Ca^2+^]_i_ and cell membrane potential in endogenous mitochondria-depleted SMCs by transplanted mitochondria **a-f.** Representative curves of hypoxia-triggered [Ca^2+^]_i_ responses in a control PASMC (**a**), PASMC cultured with pyruvate (Py) and uridine (Ur) (**b**), endogenous mitochondria-depleted PASMC by incubation with ethidium bromide (EB), Py and Ur (**c**), endogenous mitochondria-depleted PASMC transplanted with mitochondria (mito) prepared from PASMCs (PASMC-mito) (**d**) or FASMCs (FASMC-mito) (**e**), summary of **a-e** (* *p* < 0.05 *vs*. baseline, **f**). **g-l.** Representative curves of hypoxia-triggered [Ca^2+^]_i_ responses in a control FASMC (**g**), FASMC cultured with Py and Ur (**h**), endogenous mitochondria-depleted FASMC by incubation with EB, Py and Ur (**i**), endogenous mitochondria-depleted FASMC transplanted with PASMC-mito (**j**) or FASMC-mito (**k**) and summary of **g-k** (*p < 0.05 *vs*. baseline, **l**).

**Figure 4:**
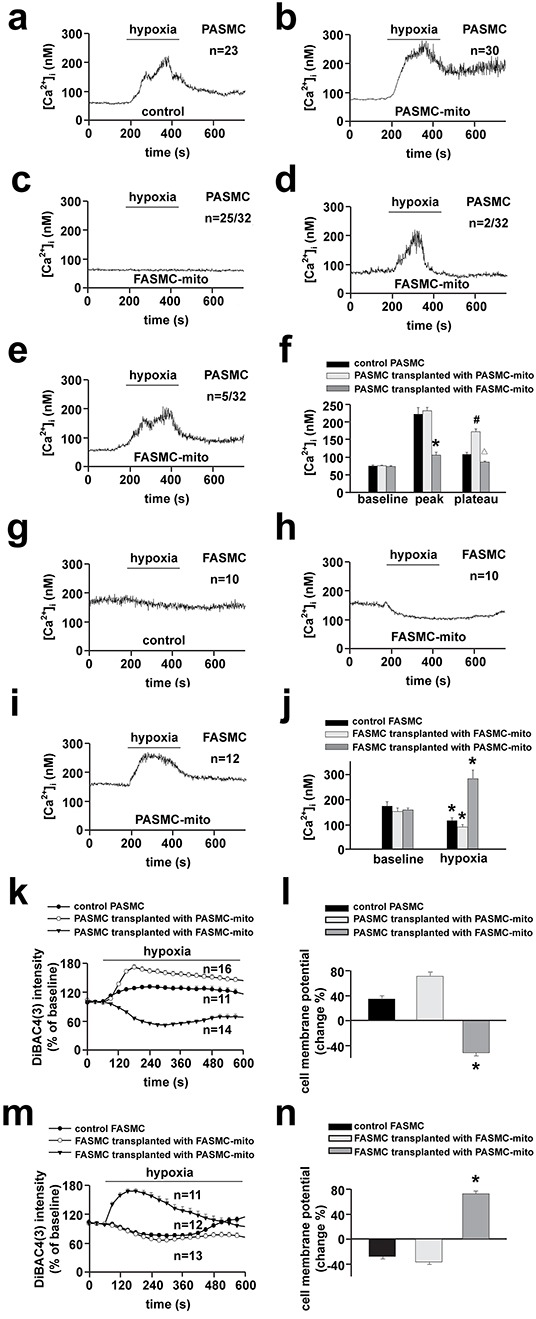
Changes of hypoxia-altered [Ca^2+^]_i_ and cell membrane potential in native SMCs by transplanted mitochondria **a-j.** Representative curves of hypoxia-triggered [Ca^2+^]_i_ responses in a control PASMC (**a**), PASMC transplanted with PASMC-mito (**b**) or FASMC-mito (**c-e**) and summary of **a-e** (* # Δ *p* < 0.05 *vs*. control, **f**); in a control FASMC (**g**), FASMC transplanted with FASMC-mito (**h**) or PASMC-mito (**i**) and summary of **g-i** (*p < 0.05 *vs*. baseline, **j**) **k-n.** Representative curves of hypoxia-triggered cell membrane potential response in a control PASMC, PASMC transplanted with PASMC-mito or FASMC-mito (**k**) and summary of maximal alterations of cell membrane potential (**l**); in a control FASMC, FASMC transplanted with FASMC-mito or PASMC-mito (**m**) and the summary (**n**). “n” indicated the cell number examined for each group.

To determine whether mitochondria are the determinants for the distinct [Ca^2+^]_i_ responses of pulmonary and systemic arterial SMCs to hypoxia, mitochondria prepared from PASMCs and FASMCs were transplanted from each other into mitochondria-depleted SMCs preparations [5]. Hypoxia stimulated a quick [Ca^2+^]_i_ elevation in native, control PASMCs (Figure 3a and 3f) with a latency of ~ 7.9 s and similarly in PASMCs incubated with pyruvate (Py) and uridine (Ur) (Figure 3b and 3f). By contrast, hypoxia did not stimulate any [Ca^2+^]_i_ elevation in mitochondria-depleted PASMCs by ethidium bromide (EB) incubation together with Py and Ur [5] (Figure 3c and 3f). The transplantation of mitochondria prepared from PASMCs into mitochondria-depleted PASMCs restored the [Ca^2+^]_i_ elevations in response to hypoxia (Figure 3d and 3f). However, the transplantation of mitochondria prepared from FASMCs into mitochondria-depleted PASMCs rendered the [Ca^2+^]_i_ decline in response to hypoxia (Figure 3e–3f). In native PASMCs transplanted with mitochondria derived from PASMCs, hypoxia stimulated [Ca^2+^]_i_ elevations (Figure 4b and 4f). In native PASMCs transplanted with mitochondria derived from FASMCs, however, hypoxia failed to trigger [Ca^2+^]_i_ signaling in 25 out of 32 cells examined (Figure 4c), induced a single [Ca^2+^]_i_ transient in another 2 (Figure 4d) and [Ca^2+^]_i_ elevations with a plateau in the remaining 5 cells (Figure 4e). Similarly, mitochondrial transplantation effectively changed hypoxia-induced [Ca^2+^]_i_ decline in FASMCs (Figure 3g–3l and 4g–4j).

Hypoxia-induced cell membrane potential depolarization in PASMCs was slightly augmented by intracellular transplantation of mitochondria derived from PASMCs (Figure 4k–4l). However, hypoxia stimulated cell membrane potential hyperpolarization in PASMCs transplanted with mitochondria derived from FASMCs (Figure 4k–4l). Hypoxia induced cell membrane potential hyperpolarization in native FASMCs and FASMCs transplanted with mitochondria derived from FASMCs (Figure 4m–4n). In FAMSCs transplanted with mitochondria derived from PASMCs, hypoxia stimulated cell membrane potential depolarization instead (Figure 4m–4n).

These experiments provide the cause-effect evidence for the determinative role of mitochondria in governing the divergent alterations of [Ca^2+^]_i_ and cell membrane potential in pulmonary and systemic arterial SMCs in response to hypoxia.

### Mitochondrial transplantation on acute hypoxia-induced vascular responses *in vitro*

**Figure 5:**
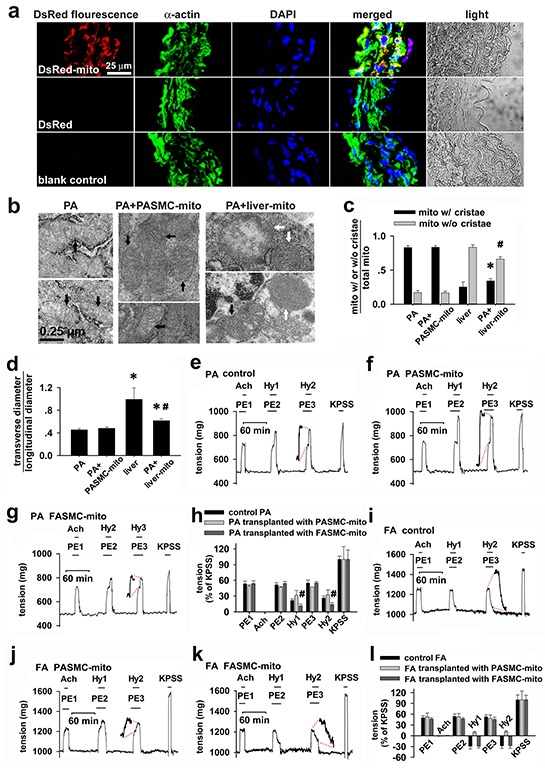
Changes of hypoxia-induced vascular responses *in vitro* by transplanted mitochondria **a.** Immunocytochemical stainings of isolated pulmonary artery in DsRed-labeled mitochondria (upper), DsRed- (middle) incubated preparations and control (lower) showing DAPI (blue), smooth muscle cell marker α-actin (green), DsRed (red), overlap of the above three and light field (n=3 for each). **b-d.** Electron micrographs showing mitochondria (mito) in SMCs in isolated control pulmonary artery (PA, left, **b**) and PA after incubating with PASMC-derived mitochondria (PASMC-mito) (black arrows, middle, **b**), mixture of mitochondria with distinct morphology in SMCs (black and white arrows, right, **b**) in isolated PA after incubation with mitochondria prepared from Wilson's rat liver (liver-mito) and quantitation of two shapes of mitochondria (* # *p* < 0.05 *vs.* mito with/without cristae in PASMCs respectively, **c**) as well as their ratio of width to length (* *p* < 0.05 *vs.* PASMCs, #*p* < 0.05 *vs.* liver, **d**). Quantitation obtained from 106, 129, 232 and 121 mitochondria of 25, 21, 20 and 23 cells from 3-4 separate vessel/liver preparations for PA, PA incubated with PASMC-mito, Wilson's liver and PA incubated with the liver-mito, respectively. **e-h.** Alterations of isometric tension in endothelium-denuded, phenylephrine (PE)-preconstricted PA rings in sequential exposures to acetylcholine (Ach), hypoxia (Hy1 and Hy2) and KPSS containing 80 mM K^+^ (the equimolar substitution of Na^+^ by K^+^) for control PA (n=8, **e**), PA transplanted with mitochondria prepared from PASMCs (PASMC-mito) (n=3, **f**) or FASMCs (FASMC-mito) (n=5, **g**) and summary of **e-g** (# *p* < 0.01 *vs*. control or PASMC-mito, respectively. **h**). **i-l.** Alterations of isometric tension in endothelium-denuded, phenylephrine (PE)-preconstricted femoral artery (FA) rings in sequential exposures to Ach, hypoxia (Hy1 and Hy2) and KPSS for control FA (n=6, **i**), FA transplanted with PASMC-mito (n=3, **j**) or FASMC-mito (n=3, **k**) and summary of **i-k (l**).

The immunohistochemical stainings employing antibody against DsRed or SMC specific α-actin showed the overlapping of DsRed with α-actin in PAs strips incubated with DsRed-labeled mitochondria, not in PAs strips incubated with 0.76 μg/ml DsRed, indicating distribution of DsdRed-labeled mitochondria within PASMCs (Figure 5a).

Consistent with those in PASMCs in culture (Figure 1c, right), mitochondria in PASMCs in PAs strips incubated with the Wilson's disease liver mitochondria were also a mixture of morphological characteristics or shapes (Figure 5b, right and Figure 5c–5d).

Endothelium-removed PAs rings constricted while each hypoxic challenge was applied to control preparations (Figure 5e and 5h). The hypoxia-induced vasoconstriction was slightly augmented in PAs rings transplanted with mitochondria from PASMCs (Figure 5f and 5h), whereas significantly attenuated in those transplanted with mitochondria from FASMCs (Figure 5g–5h). The endothelium-removed rings of femoral arteries (FAs) relaxed in response to each hypoxic challenge (Figure 5i and 5l). Instead, hypoxia induced constriction in FAs rings transplanted with mitochondria from PASMC (Figure 5j and 5l).

Respiratory Functional evaluation, measurements of ROS generation and mitochondrial membrane potential in isolated mitochondria sorted and recovered from PAs by flow cytometry after transplantation of exogenous mitochondria indicated that exogenous mitochondria retained their heterogeneous properties (Supplementary Figure S3).

These results indicate the pivotal role of mitochondria in determining vascular responses to hypoxia and the potential application of exogenous mitochondria in attenuating HPV.

### Intrinsic diversity between mitochondria of PASMCs and FASMCs

**Figure 6:**
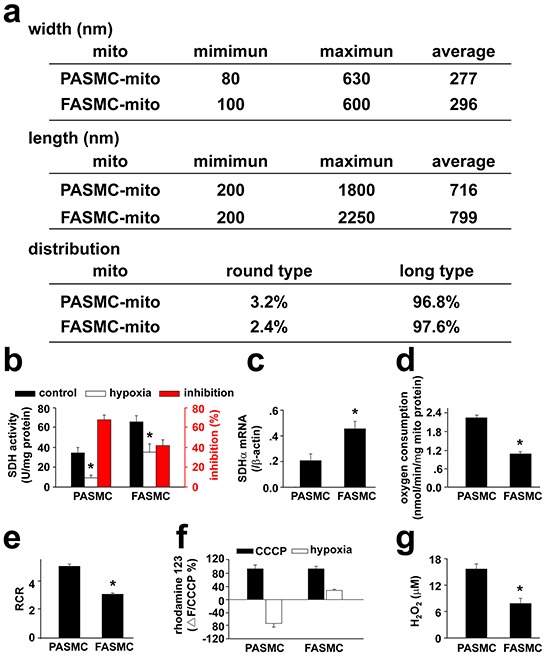
Comparison of mitochondria in pulmonary artery smooth muscle cells and femoral artery smooth muscle cells **a.** The ultrastructual morphology of mitochondria: the width and length were obtained from 221 mitochondria in 49 separate pulmonary artery smooth muscle cells (PASMCs) and 93 mitochondria in 13 separate femoral artery smooth muscle cells (FASMCs), respectively. The ratio of length to width between 1-1.25 or above 1.25 was used to define round or long type mitochondria, respectively. **b.** mitochondrial succinic dehydrogenase activity (SDH): the freshly isolated mitochondria from PASMCs and FASMCs were quantified by protein determination using BCA assay. The SDH activity was measured by monitoring SDH-catalyzed reduction of FAD to FADH, which was coupled to the reduction of 2, 6-dichlorophenolindophenol (* *p* < 0.05, n=6 for each). **c.** SDH gene expression: The mRNA levels of SDH in PASMCs and FASMCs were quantified by real-time PCR with primers targeting on the SDH sub unit α (SDHα) and normalized by β-actin, respectively (* *p* < 0.05, n=3 for each). **d.** oxygen consumption: the oxygen consumption in mitochondria isolated from PASMCs and FASMCs was determined using a Clark-type oxygen meter and electrode system (*p < 0.05, n=4 for each). **e.** respiratory control ratio (RCR): the RCR was determined in mitochondrial suspension with a Clark-type oxygen meter and electrode system and a Mitochondria RCR Assay Kit (*p < 0.05, n=3 for each). **f.** alterations in mitochondrial membrane potential (MMP) in response to hypoxia: Rhodamine 123 was loaded into PASMCs and FASMCs and the alterations in rhodamine123 fluorescence in response to hypoxia were normalized to the changes induced by CCCP (n=10 for PASMCs or 11 for FASMCs). **g.** generation of reactive oxygen species (ROS) in response to hypoxia: ROS generation from hypoxia-stimulated PASMCs and FASMCs was monitored with DCFDA and calibrated using a series of extracellular H_2_O_2_ (n=22 for PASMCs or 15 for FASMCs).

The isolated mitochondria of FASMCs exhibited higher intrinsic activity and gene expression level of succinic dehydrogenase, lower respiratory function (as indexed by oxygen consumption and respiratory control ratio) under normoxic condition and produced lower amount of reactive oxygen species in response to hypoxia as compared to the mitochondria of PASMCs (Figure 6).

### Dose-dependent alterations in [Ca^2+^]_i_, cell membrane potential and vascular tension in response to H_2_O_2_

**Figure 7:**
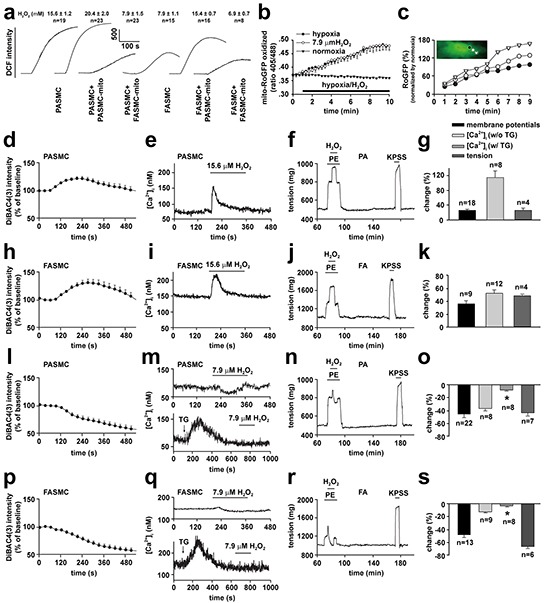
Dose-dependent alterations in cell membrane potential, [Ca^2+^]_i_ and vascular tension in exposure to H_2_O_2_ **a-c.** ROS generation in hypoxia-exposed pulmonary (PASMCs) and femoral artery smooth muscle cells (FASMCs). Representative alterations of DCFDA intensity in PASMCs and FASMCs in exposure to hypoxia and calibrated H_2_O_2_ levels (**a**). Quantified time-course changes in RoGFP intensity ratios from excitation of 405 nm versus 488 nm with emission of 515 nm from FASMCs exposed to hypoxia, 7.9 μM H_2_O_2_ or normoxia (n= 4 for each, **b**). (**c**). Representative and quantitative estimation of localized alterations of cytosol RoGFP fluorescence in the area close to cell membrane, the middle and the one close to nucleus from a PASMC upon exposure to hypoxia (n=3 for each, **c**). **d-s.** Representative curves and averaged changes of DiBAC4 (3) fluorescence for cell membrane potential, fura-2 fluorescence for [Ca^2+^]_i_ in smooth muscle cells and tension of artery rings in response to 15.6 μM (**d-k**) or 7.9 μM H_2_O_2_ (**l-s**) in PASMCs (**d**, **e**, **g** and **l**, **m**, **o**) and pulmonary artery (**PA**, **f-g** and **n-o**), or in FASMCs (**h**, **i**, **k** and **p**, **q**, **s**) and femoral artery (**FA**, **j-k** and **r-s**); in PASMCs without or with treatment of 1 μM TG (upper and lower, **m**), or in FASMCs without or with treatment of 1 μM TG (upper and lower, **q**). The changes of membrane potential and [Ca^2+^]_i_ were compared with their baseline, respectively and the tension were normalized by the response induced by 80 mM K^+^ (KPSS), while “n” indicating the number of separate experiments for each.

**Figure 8:**
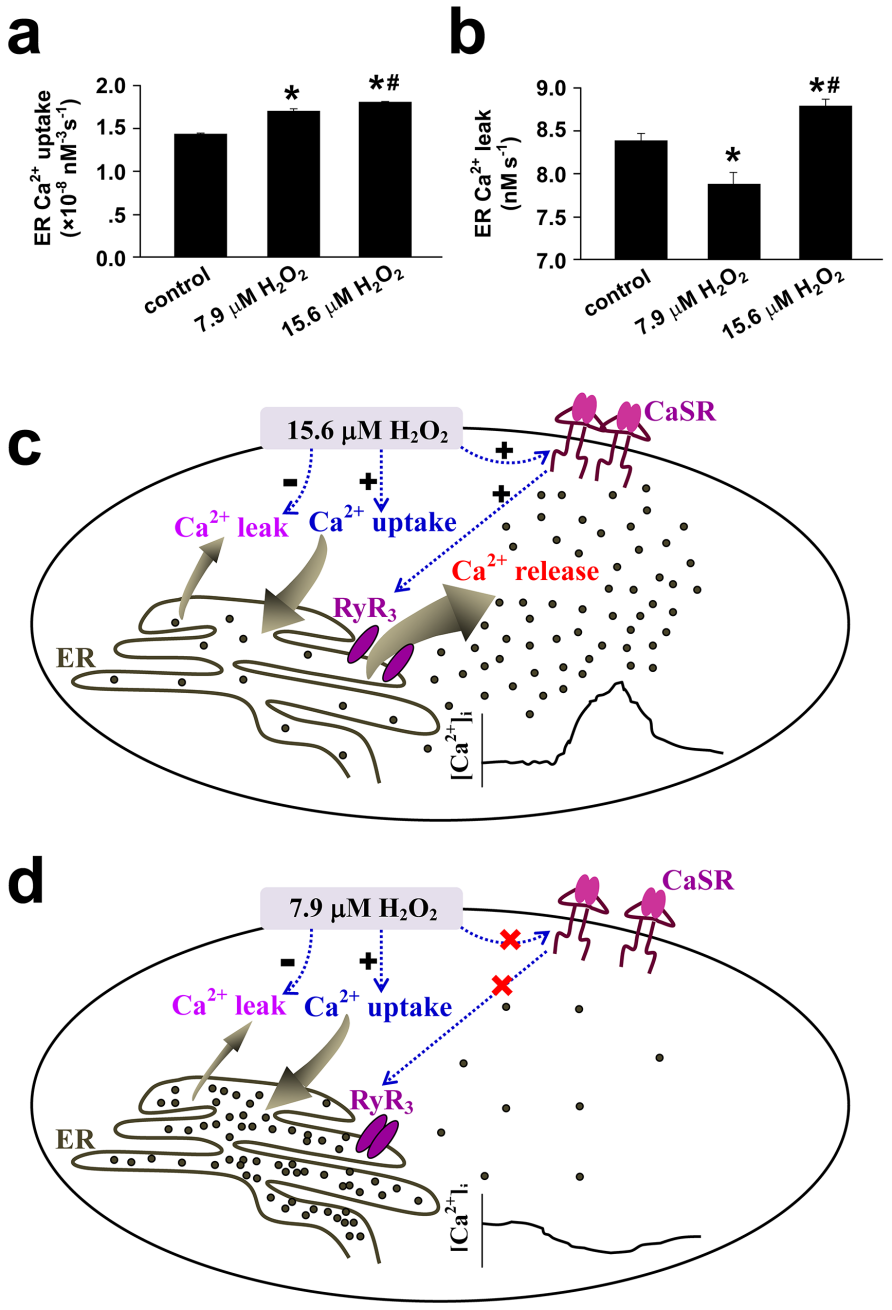
Mechanisms underlying the opposite effects of 15.6 vs 7.9 μM H_2_O_2_ on the regulation of [Ca^2+^]_i_ in vascular smooth muscle cells **a-b.** The endoplasmic reticulum (ER) Ca^2+^ uptake(**a**) and Ca^2+^ leak(**b**) were estimated from H_2_O_2_-induced alterations in [Ca^2+^]_i_ in vascular smooth muscle cells following the established techniques and the equation of d[Ca^2+^]_i_d*t* = *A*[Ca^2+^]_i_*^4^* − *L*, where *A* and *L* reflect the rate of ER Ca^2+^ uptake and the Ca^2+^ leak, respectively(n=9 for control and 7.9 μM H_2_O_2_, n=12 for 15.6 μM H_2_O_2_, *#*p* <0.01 *vs*. control or 7.9 μM H_2_O_2_, respectively). **c.** 15.6 μM H_2_O_2_-induced sensitization and activation of extracellular calcium-sensing receptor (CaSR) results in Ca^2+^ release from ER via ryanodine receptor3 (RyR_3_). Although 15.6 μM H_2_O_2_ increases the ER Ca^2+^ uptake and passive Ca^2+^ leak from ER, the net effects of 15.6 μM H_2_O_2_ is to elevate [Ca^2+^]_i_. **d.** 7.9 μM H_2_O_2_ fails to sensitize CaSR, but increases ER Ca^2+^ uptake and inhibits passive Ca^2+^ leak from ER and therefore results in a decline in [Ca^2+^]_i_.

To understand how transplanted mitochondria induced the above changes in cellular behaviors of SMCs in response to hypoxia, hypoxia-altered generation of reactive oxygen species (ROS) [4, 5, 8] was evaluated in SMCs of different preparations. The hypoxia-altered ROS generation from SMCs has been well shown to be pivotal in mediating hypoxia-changed cellular behaviors such as [Ca^2+^]_i_ signaling and constriction or relaxation in coronary artery [14, 15] and PAs [3, 4, 5, 8], respectively. Hypoxia increased DCF fluorescence in FASMCs (Figure 7a). The quantitative estimation of DCF fluorescence and the verification by RoGFP revealed an equivalent level of 7.9 μM H_2_O_2_ generation from hypoxia-stimulated FASMCs (Figure 7a–7b), which was lower than ROS level of 15.6 μM H_2_O_2_ in hypoxia-stimulated PASMCs [5]. Hypoxia-stimulated ROS production was slightly increased in PASMCs by transplantation with mitochondria derived from PASMCs. Hypoxia-stimulated ROS generation was obviously decreased, to a level similar to hypoxia-stimulated native FASMCs, in PASMCs by transplantation with mitochondria derived from FASMCs (Figure 7a). Transplantation of mitochondria derived from PASMCs increased ROS generation from hypoxia-stimulated FASMCs, however, hypoxia stimulated similar ROS generation from FASMCs transplanted with mitochondria derived from FASMCs (Figure 7a). Hypoxia stimulated ROS generation quickly in both PASMCs and FASMCs with the similar latencies of ~ 2 s and the most prominent alteration of ROS seemed localized at subcellular areas close to cell membrane (Figure 7c). In PASMCs, the activity of succinic dehydrogenase (SDH), a mitochondrial enzyme whose inhibition by hypoxia was critically involved in hypoxia-stimulated ROS generation [3, 4] in PASMCs was ~ 50% of SDH activity in FASMCs (Figure 6b). 15.6 μM H_2_O_2_ triggered cell membrane potential depolarization, [Ca^2+^]_i_ elevation and constriction in both PASMCs/PAs rings (Figure 7d–7g) and FASMCs/FAs preparations (Figure 7h–7k). In contrary, 7.9 μM H_2_O_2_ induced cell membrane potential hyperpolarization, [Ca^2+^]_i_ decline and relaxation in both PASMCs/PAs rings (Figure 7l–7o) and FASMCs/FAs rings (Figure 7p–7s). Further estimation of Ca^2+^ movement across endoplasmic reticulum (ER) showed that 7.9 μM H_2_O_2_ significantly increased ER Ca^2+^ uptake and decreased Ca^2+^ leak, a non-specific, constitutive or passive Ca^2+^ leak from ER. However, 15.6 μM H_2_O_2_ increased both ER Ca^2+^ uptake and passive Ca^2+^ leak (Figure 8a–8b).

To address what numbers of exogenous mitochondria were needed for overwhelming the function of the endogenous mitochondria, the numbers of intracellularly transplanted mitochondria were purposely controlled by incubating PASMCs with 2.25×10^8^/ml FASMC-mito for 6, 12, 18 and 24 hours, respectively. This was based on the incubation time period-dependent dynamical transplantation of exogenous mitochondria into PASMCs (Figure 2e). These PASMCs were then exposed to hypoxia and the levels of ROS generation was measured and quantified as the index of overwhelming the function of the endogenous mitochondria. The relationship curve between the number of intracellularly transplanted FASMC-mito and ROS levels was statistically aggregated and generated (Supplementary Figure S4). Based on our previous finding that 13 μM H_2_O_2_ failed to trigger Ca^2+^ signaling and pulmonary vasoconstriction [5], the threshold or minimum amount of intracellular FASMC-mito necessary for overwhelming the function of the endogenous mitochondria was finally determined at ~ 256 per PASMC.

These results suggest that the difference between mitochondria of PASMCs and FASMCs lies in their capability to generate ROS in response to hypoxia and the levels of ROS determine SMC behaviors in the pattern of cell membrane potential depolarization, [Ca^2+^]_i_ elevation and constriction or cell membrane potential hyperpolarization, [Ca^2+^]_i_ decline and relaxation.

## DISCUSSION

To mechanistically determine if mitochondria indeed fulfill Koch's Postulates as the key cause or trigger of HPV [2, 5, 7, 8, 10], the current study established a way to transplant exogenous mitochondria into SMCs. The mitochondrial transplantation may be associated with the direct contact between mitochondrial membrane and cytoplasma membrane and the subsequent processes like fusion and or endocytosis/macropinocytosis (Supplementary Figure S2). The coculture of endogenous mitochondria-depleted A549ρº cells with isolated mitochondria showed no mitochondrial transfer into A549ρº in the absence of Py and Ur [16]. However, the very recent studies showed that mitochondrial transfer into cardiomyocyte or endometrium-derived mesenchymal cells can be achieved with isolated mitochondria by local injection or co-culture *in* vitro [17, 18]. The exact reason underling the distinct feasibility of mitochondrial transplantation into cells is unknown, but may be the cell type and or experimental conditions particularly the presence of Py and Ur and mitochondrial preparations.

Hypoxia-induced PAs constriction and FAs relaxation were reversed by cross transplantation of their mitochondria from each other (Figure 5). The reason seems to be the reversible effects of transplanted mitochondria on hypoxia-altered [Ca^2+^]_i_ signal and cell membrane potential (Figure 3 and 4). The depolarization of cell membrane potential is expected to activate voltage-dependent calcium channels (VDCC) including L-type VDCC in PAs as previously reported and recently summarized [19]. However, the exact role of L-type VDCC is complicated, since the opening of L-type of VDCC can allow the influx of extracellular Ca^2+^ and can also induce the release of intracellular Ca^2+^ from ER [15]. Hypoxia-induced elevation or decline in [Ca^2+^]_i_ or Ca^2+^ channel activity including L-type VDCC in SMCs has been broadly accepted to be pivotal in initiating vascular constriction or relaxation in response to hypoxia, respectively [4, 5, 15, 19], and to critically lie in the redox status of mitochondria under hypoxia [2, 3, 4, 5, 8, 20]. The results of the current study (Figure 7) and a very recent one of ours [5] are generally consistent with those showing that SMCs of pulmonary artery [4, 8] and systemic artery [8, 14, 20] both produced ROS under hypoxia. Whereas, the quantitative estimations of ROS level from our studies further demonstrated that mitochondria in PASMCs produced more amount of ROS than FASMCs under hypoxia, equivalent 15.6 μM H_2_O_2_ in PASMCs vs. 7.9 μM H_2_O_2_ in FAMSCs (Figure 7). H_2_O_2_ induced cell membrane potential hyperpolarization, decline in [Ca^2+^]_i_ and vasorelaxation as well as cell membrane potential depolarization, elevation in [Ca^2+^]_i_ and vasoconstriction in both PA and FA depending on low (7.9 μM) or high (15.6 μM) amount of H_2_O_2_ applied (Figure 7). The above results are in agreement with a previous investigation showing that the dosage of H_2_O_2_ employed in experiments appeared to determine whether H_2_O_2_ induced constriction or relaxation [21]. In fact, H_2_O_2_ induced both relaxation and constriction in many vascular beds in a variety of species [21, 22, 23, 24], sometimes in the same vesse [21, 22, 24]. The distinct effects on [Ca^2+^]_i_ induced by high verseus low H_2_O_2_ may be due to their capability in sensitizing the extracellular calcium-sensing receptor (CaSR) [5] and in controlling the ER Ca^2+^ uptake and leak (Figure 8). 15.6 μM H_2_O_2_-induced sensitization and the subsequent activation of CaSR by extracellular Ca^2+^ resulted in intracellular Ca^2+^ release from ryanodine receptor3 as well as STIM1-controlled extracellular Ca^2+^ influx [5]. Although 15.6 μM H_2_O_2_ increased the ER Ca^2+^ uptake and passive Ca^2+^ leak (Figure 8), the net effects of 15.6 μM H_2_O_2_ was to elevate [Ca^2+^]_i_ (Figure 8). By contrast, 7.9 μM H_2_O_2_, below the threshold level of 13-15.6 μM for CaSR sensitization [5], failed to sensitize CaSR, but increased ER Ca^2+^ uptake and inhibited passive Ca^2+^ leak from ER (Figure 8) and therefore resulted in a decline in [Ca^2+^]_i_ (Figure 8). In support of our findings about H_2_O_2_-controlled ER Ca^2+^ uptake and Ca^2+^ leak, superoxide (O_2_^−.^) was reported to increase ATPase activity and to accelerate Ca^2+^ uptake in microsome/ER of PASMCs [25]. H_2_O_2_ at high micromolar concentrations (≥ 100 μM) has been shown to increase Ca^2+^ leak in skeletal muscle [26]. Thus the molecular mechanism underlying the distinct vascular responses to hypoxia between pulmonary and systemic circulation seems to be the more potent capability of mitochondria to produce ROS under hypoxia in PASMCs than FASMCs. And the transplanted mitochondria derived from FASMCs possibly work through limiting hypoxia-induced H_2_O_2_ generation in PASMCs to a level (Figure 7) lower than the threshold for CaSR [5]. The lower intrinsic activity of SDH maintains higher amount of oxygen consumption in mitochondria of PASMCs than FASMCs under normoxic condition (Figure 6). Hypoxia, however, decreased or lowered the activity of mitochondrial SDH [3, 4]. The further lowered SDH activity (Figure 6) and the higher amount of oxygen consumption in PASMCs under hypoxic condition would be expected to result in the accumulation of more free electrons from the electron transport chain and the subsequent production of high amount of ROS than FASMCs (Figure 6).

The intracellular distribution of mitochondria is not uniform and profoundly controls cellular behavior [1, 27, 28], including [Ca^2+^]_i_ signaling [27, 28]. The mitochondria localized in areas close to cytoplasma membrane were suggested to sense hypoxia and mediate downstream events in PASMCs [1]. In the current study, the transplanted mitochondria were found to be primarily localized in the areas close to cytoplasma membrane and this can partially explain why the hypoxia-induced cellular signaling and vascular responses were changed by exogenous mitochondria while endogenous mitochondria still retained in the cells. The other possible explanation for the altered behavior of SMCs by exogenous mitochondria can be their fusion with endogenous counterpart [9]. The supportive information about fusion of exogenous mitochondria with their endogenous counterparts in the current studies includes the fluorescence overlapping of exogenous and endogenous mitochondria in live cell imagings (Figure 1), direct contact and or fusion of the exogenous mitochondria (prepared from femoral artery smooth muscle cells or Wilson's liver) with endogenous one under ultra-structural examination (Figure 1) and co-existence of fluorescent labelings of exogenous and endogenous mitochondria sorted and recovered by flow cytometry (Figure 2).

Hypoxic vasoreaction is (patho-)physiologically important in many tissues in addition to vessels. The findings of the current study may therefore be suggestive for our understanding and changing hypoxia-induced events *in vitro*. This study also warrants further studies *in vivo* in exploring a novel strategy of mitochondrial transplantation for the treatment of hypoxic pulmonary hypertension.

## MATERIALS AND METHODS

### Study design

Following the policies of institute animal care and ethics, animal experiments with the fewest number of rats (typically ≤ 6) were designed and conducted to obtain the required data. The current study aimed to determine whether exogenous mitochondria can be transplanted into smooth muscle cells (SMCs) and pulmonary arteries (PAs); then whether the transplantation of exogenous mitochondria alters the cellular behaviors of vascular SMCs in response to hypoxia; and finally to reveal the molecular mechanisms underlying the role of transplanted mitochondria in altering the cellular responses to hypoxia.

The transplantation of exogenous mitochondria was evaluated and quantified by live cell imaging using mitochondria-targeted DsRed as tracer. The success of mitochondrial transplantation into cultured cells and pulmonary arteries *in vitro* was confirmed by immunocyto-/histochemical stainings using antibody against the tracer, flow cytometry sorting and recovery, the employment of Wilson's rat liver mitochondria with distinct ultrastructure, and the adoption of a newly-established technique using engineered ascorbate peroxidase (APEX) as a genetic tag to label mitochondria for ultra-structural visualization in cell preparations. The internalization or intracellular delivery of intact mitochondria rather than merely endocytosed fluorescent tracer was further verified using DeRed, GFP and APEX protein. The endocytosis-associated pathways were explored for the mechanism underlying the intracellular transplantation or internalization of intact mitochondria.

The mitochondrial function were assessed by measuring their respiratory control ratio, oxygen consumption, reactive oxygen species generation and alteration of mitochondrial membrane potential (MMP) in mitochondrial suspension isolated *in vitro* or recovered by flow cytometry after transplantation.

Cellular behaviors in cell membrane potential, [Ca^2+^]_i_ signaling and MMP were assessed by live cell fluorescent imaging of DiBAC4 (3), Fura-2 and rhodamine 123, respectively. The behavior of constriction or relaxation of SMCs in response to hypoxia was quantified by isometric tension measurement of the rings of intralobar pulmonary artery and femoral artery.

To reveal the mechanism underlying the role of transplanted mitochondria, hypoxia-altered cellular oxidative status was monitored by quantification of reactive oxygen species/hydrogen peroxide (ROS/H_2_O_2_) generation, spatial distributions within SMCs in response to hypoxia with live cell imaging of DCFDA and RoGFP fluorescence. The potential role of mitochondrial succinic dehydrogenase in the above process and the (dis)ability of low or high level of H_2_O_2_ in activating extracellular Ca^2+^-sensing receptor were explored. The cultured SMCs and isolated pulmonaries artery and femoral arteries were exposed to different dosages of H_2_O_2_ to mimic hypoxia-induced oxidant stress at different levels, and cell membrane potential, [Ca^2+^]_i_ signaling as well as isometric tension were selected as endpoints.

### Ethical approval

All our studies using Sprague-Dawley (SD) rats were approved by the Institutional Animal Care and Use Committee of the Tongji Medical College, Huazhong University of Science and Technology, and performed in accordance with the National Institutes of Health Guide for the Care and Use of Laboratory Animals.

All detailed material and methods are provided in the Online Supplement as an attachment.

## SUPPLEMENTARY MATERIALS FIGURES


