# Supplementary material for: Mitochondrial transplantation attenuates hypoxic pulmonary vasoconstriction
Source: Oncotarget. 2016 Apr 21;7(21):31284–98. doi: 10.18632/oncotarget.8893 (PMC5058756; doi:10.18632/oncotarget.8893)
Supplement: Supplementary file 1 [file oncotarget-07-31284-s001.pdf]

SUPPLEMENTARY METHODS

Anaesthesia and euthanasia

SD rats were deeply anesthetized by an intraperitoneal injection (I.P.) of urethane (1.2 g/kg in phosphate-buffered saline, PBS). When the sufficient status of anaesthesia was confirmed by negative paw pinch test, the femoral arteries and lung tissues were excised quickly for the isolation of pulmonary arteries and the subsequent culture of smooth muscle cells, the measurement of isometric tension, immunohistochemical analysis and electron microscopy study.

Cell culture

The rat smooth muscle cells (SMCs) were primarily cultured from explants of the third branches of pulmonary artery or femoral artery with Dulbecco's Modified Eagle Medium (DMEM) containing 10% fetal bovine serum (FBS) [1, 2]. The SMCs in 1 or 2 passages with ~ 70% confluence were used in experiments. To deplete mitochondrial DNA, SMCs were treated with 110 μg/ml sodium pyruvate, 50 μg/ml uridine and 200 ng/ml ethidium bromide as fully described before [1, 2]. The SMCs employed for each kind of experiments were cultured from ≥ 3 rats.

Mitochondrial labeling with florescence and preparations from SMCs

To label mitochondria, mitochondria-targeted expression vector of DsRed2-Mito or pAcGFP1-Mito (Clontech) was transfected into SMCs using Lipofectamine 2000 (Invitrogen) as reported before [1, 2]. Briefly, SMC monolayers grown in 60-mm dishes were used for transfection with 4 μg plasmid in 0.25 ml opti-MEM containing 10 μl Lipofectamine 2000 at 37°C and 5% CO_2. After 6 hours, culture medium was changed into DMEM containing 10% FBS. DsRed and GFP protein were obtained from Clontech.

Mitochondria were isolated from cultured SMCs by a modification of the previously described method for studying biogenetically and functionally competent mitochondria [3, 4, 5] as follows. The whole purification process was performed on ice or at 4°C. The cells in exponential growth were trypsinized and washed by buffer containing 1.0 Tris–HCl, 130 NaCl, 5.0 KCl, 7.5 $MgCl_2$ (mM, pH 7.0), fragmented in 50% of the packed cell volume of 3.5 Tris–HCl, 2.0 NaCl and 0.5 $MgCl_2$ (mM, pH 7.8) using a homogenizer. The cell homogenate was quickly blended with 1:9 of the packed cell volume of 350 Tris–HCl, 200 NaCl, 50 $MgCl_2$ (mM, pH 7.8) and centrifuged for 5 min at 1500 g for pelleting unbroken whole cells, cell debris and free nuclei. The aspirated supernatant was recentrifuged under the above settings. The final supernatant was then partitioned into eppendorf tubes and centrifuged for 1 min at ~ 15, 000 g. The pellets of mitochondria were washed once by 35 Tris–HCl, 20 NaCl, 5 $MgCl_2$ (mM, pH 7.8), twice by 10 Tris–HCl, 1 EDTA, 320 saccharose (mM, pH 7.4), then re-suspended in the appropriate incubation medium. Quantification of isolated mitochondria was performed using MitoTracker Green (Invitrogen) or employing the BCA protocol (Pierce).

Mitochondrial numbering

The mitochondrial number was viably determined by counting an aliquot of isolated mitochondria labeled with MitoTracker Green (Invitrogen) under a fluorescent microscope (OLYMPUS IX-70). To label mitochondria, the isolated mitochondria were incubated with 0.1 μM MitoTracker Green at 37ºC for 1 hour followed by complete wash by 35 Tris–HCl, 20 NaCl, 5 $MgCl_2$ (mM, pH 7.8) for three times, and finally re-suspended in 100 μL the appropriate incubation buffer at room temperature. 10 μl aliquots of labeled mitochondria were loaded onto a cytometer. The cytometer was then covered with a glass coverslip and invertedly mounted on the stage of a modified Olympus inverted epifluorescence microscope (OLYMPUS IX-70). The glass coverslip "sealed" the 10 μL aliquot within the cytometer and thus prevented it from leaking or dropping when invertedly mounted on the stage of microscope. The mitochondria were visualized with fluorescence excitation at 488 nm and emission at 515±10 nm wavelength, respectively. The capture and analysis of MitoTracker Green images were performed using the software Invivo 3 (Media Cybernetics, Rockville, MD) and Image-pro Analyzer 6.2 (Media Cybernetics), respectively. A light field image of the same area was obtained for determining the counting area for each measurement.

To validate the accuracy of the above mitochondrial counting, flow cytometry was performed to determine the number of mitochondria isolated from cells. 300 μL aliquots of MitoTrack Green-labeled mitochondria were processed on a FACS flow cytometer (BD FACSAria II, Becton, Dickinson and Company, USA) and subsequent analysis using a BD FACSDiva software. The unlabeled mitochondria were used a negative control to set up voltages of forward scatter (FSC) and side scatter (SSC) at 220 and 346v, respectively. The wavelengths for detecting MitoTrack Green fluorescence were 488 nm and 530±30 nm for excitation and emission, respectively. The mitochondrial number determined by flow cytometry (Nf) was ~ 19% more than the number counted by cytometer (Nc) from the same mitochondrial

preparations (Nf:Nc=1.087:0.911, n=3) and regression analysis confirmed a significant correlation between the two methods (Nf=0.7665×Nc+576507, r^2=0.99).

Separation and recovery of transplanted mitochondria

To separate and recover exogenous mitochondria after their transplantation into pulmonary artery smooth muscle cells (PASMCs), the endogenous mitochondria of PASMCs were pre-labeled with GFP and then the PASMCs were incubated with 2.35×10^8/ml DsRed-labeled exogenous mitochondria prepared from femoral artery smooth muscle cells (FASMCs) at 37ºC for 24 hours. Then mitochondria were isolated from ~ 2.5×10^5 PASMCs, suspended in 500 µl Tris-buffer containing 320 saccharose, 1 EDTA, 10 Tris-Base (mM, pH 7.4), BSA (0.1% wt/vol). 300 µl of the mitochondrial suspension were processed on a FACS flow cytometer (BD FACSAria II, Becton, Dickinson and Company, USA) and subsequently analyzed using a BD FACSDiva software. The unlabeled mitochondria were used a negative control to set up voltages of forward scatter (FSC) and side scatter (SSC) at 220 and 346v, respectively. The wavelengths for detecting GFP and DsRed fluorescence were 488 nm and 530±30 nm, or 554 nm and 585±15 nm for excitation and emission, respectively.

To recover exogenous mitochondria after their transplantation into pulmonary arteries, artery rings were incubated with DsRed-labeled mitochondria suspension (2.25×10^8/ml) in physiological salt solution bubbling with oxygen at 4°C overnight. The mitochondria isolated from the pulmonary arteries were suspended in 500 µl Tris-buffer and 300 µl of the mitochondrial suspension were processed on a FACS flow cytometer for the recovery of the endogenous mitochondria and DsRed-labeled mitochondria using the above settings for DsRed fluorescence.

$[Ca^{2+}]_i$, reactive oxygen species (ROS) measurement and hypoxic stimulation in smooth muscle cells

$[Ca^{2+}]_i$ was measurement using Fura-2, ROS monitoring and quantitative estimation using DCFDA and RoGFP as well as hypoxic stimulation in SMCs were performed in saline without any serum or peroxidase at room temperature as recently described in detail [2].

The H_2O_2 concentration was calibrated by exploiting the oxidation of DCFDA as conducted in our recent study [2]. The similar method has also been employed successfully in other laboratories [6, 7]. Four independent experiments were performed for each of a series of extracellular H_2O_2 concentrations of 0.1, 1, 10, 20, 30, 50, 100 µM applied to SMCs and the resulting oxidation of DCFDA were plotted again the concentration of H_2O_2 to generate the calibration curve and equation.

Hydrogen peroxide (H_2O_2)-affected endoplasmic reticulum (ER) Ca^{2+} uptake and Ca^{2+} leak were estimated from H_2O_2-induced alterations in $[Ca^{2+}]_i$ following the well-established techniques and the equation of $\mathbf{d[Ca^{2+}]_i}$ $\mathbf{d}\boldsymbol{t} = \boldsymbol{A}\mathbf{[Ca^{2+}]_i}^{\boldsymbol{4}} - \boldsymbol{L}$, where $\boldsymbol{A}$ and $\boldsymbol{L}$ reflect the rate of ER Ca^{2+} uptake and Ca^{2+} leak, respectively [8, 9].

The RoGFP was also used in the current study to monitor ROS alteration in the cytosol including subcellular changes. The RoGFP, a probe more specific for H_2O_2/oxidants than NO [10] was generated following the technical procedures initiated from Tsien's lab in 2004 [10]. The RoGFP-expression vector was produced from pEGFP-N1 plasmid (Clontech, CA) by the incorporation of the mutations of C48S, Q80R, S147C and Q204C into the GFP-encoding area [10] with the QuikChange Multi Site-directed Mutagenesis Kit (Stratagene, CA). Sequencing in forward and reverse directions in more than two separate clones was conducted at ABI 3730 Automated Sequencer (Applied Biosystems, CA) to verify the correct mutations. The RoGFP images in the cytosol of PASMCs were obtained using an oil lens (OLYMPUS uplansApo 60X/1.35) under an Olympus inverted laser scanning confocal microscope (OLYMPUS IX-70) with Diode (FV5-LD405) or Argon laser (FV5-LA-AR-230) excitation of 405 or 488 nm and a band pass filter of 515 ± 10 nm emission corresponding to the status of oxidation and reduction, respectively.

For acute hypoxia [2], the saline buffer under normoxia in the chamber was rapidly exchanged to hypoxic saline buffer via a sealed flow setup. The hypoxic saline buffer was prepared ready through blistering 100% N_2 continuously before experiments. The oxygen pressure (PO_2) in saline buffer in the bath was continuously monitored using an ISO_2 oxygen electrode and meter (World Precision Instrument). As indexed by a fast decline in PO_2 from ~ 150 to ~ 20 mmHg, hypoxia was achieved within ~ 10 sec.

Cell and mitochondrial membrane potential measurement

To measure cell membrane potential, SMCs were pre-loaded with 5 mM DiBAC4 (3) at 37°C for 20 min and the alterations of their fluorescence before and after hypoxia were observed on the confocal microscope with excitation at 488 nm and emission at 525 nm, respectively. To measure mitochondrial membrane potential (MMP) in cultured cells [11, 12], SMCs were loaded with 6 µM rhodamine 123 in PBS at 37°C for 40 min in darkness and then the excess dye was removed by complete wash with rhodamine 123-free PBS for three times, hypoxia-altered rhodamine 123 fluorescence obtained on the confocal microscope with excitation at 480 nm and emission at 515

nm, respectively, was normalized to 10 µM CCCP. The specific staining of mitochondria by 6 µM rhodamine was verified by >89% overlapping of rhodamine fluorescent imaging with DsRed in PASMCs pre-labeled by mitochondria-targeted probe, the DsRed2-Mito plasmid (Clontech).

Assessment of mitochondrial respiratory function

Theoxygen consumption in suspensions of SMCs and isolated mitochondria was determined on a Clark-type oxygen meter and electrode system (Oxytherm, UK) in a sealed glass chamber. SMCs or mitochondria suspended in 2 ml of DMEM culture medium were placed in the chamber with a magnetic stirrer at 37°C. Theoxygen consumption is defined as nanomols O_2 consumed per minute per µg mitochondrial protein or 10^6 cells. The respiratory control ratio (RCR)in isolated mitochondria was measured using the Clark-type oxygen electrode system and a Mitochondria RCR Assay Kit (Genmed. Arlington, MA, USA) following the manufactures' procedures. Briefly, the chamber was filled with 2 ml medium containing KCl 100 mM, K_2HPO_4 10 mM, $MgCl_2$ 10 mM, EDTA 1 mM, glucose 20 mM, MOPSO 50 mM and glutamate 5 mM, sealed and incubated for 1 min, then 20 µl isolated mitochondria (0.1 mg protein/µl) were introduced into the chamber through a connected microinjector and incubated for 1 min. The respiration of mitochondria was originated by supplying the substrates of pyruvate (1.25 mM), malate (1.25 mM) and an inhibitor of ATP synthase, oligomycin (2 µM) through the microinjector, and the reaction was allowed for 2 min for the determination of State IV respiration rate. The State III respiration rate was determined before and after adding an uncoupler, carbonyl cyanide mchlorophenylhydrazone (CCCP, 3 µM) and 0.375 mM ADP. RCR is defined to be the ratio of State III versus State IV respiration rate. Triplicate measurements were performed for each separate experiment.

Measurement of force

The measurement of isometric tension of the rings of intralobar pulmonary arteries (PAs) and femoral arteries (FAs) were performed as we recently reported [2].

For mitochondria delivery, artery rings were incubated with mitochondria suspension (2.25×10^8/ml) in physiological salt solution bubbling with oxygen at 4°C overnight.

Liver of Wilson's disease rat model

The Wilson's disease model was developed by copper-loaden diet in rats [13], which were fed with food containing 1g/kg copper sulfate and water containing 0.185% copper sulfate for 8 weeks. The mitochondria in the liver of the above Wilson's disease rats were characteristic in ultrastructure including unclear and greatly swelling cristae, increased density of matrix, discrete inclusions as well as shape alterations [13], distinguishable in morphology from mitochondria in SMCs in normal rats and thus employed in the present study as one tracer to track the localization of exogenous mitochondria.

Electron microscopy and ultrastructural study

The lung tissues and pulmonary arteries from at least 3 rats for each group were fixed following Karnowsky's method [13]. The thin sections were made with an ultramicrotome, stained by OsO4, and examined on a transmission electron microscope (FEI Tecnai G2 20 TWIN) using an Olympus CCD (Cantega G2) and software (Cantega G2) with an acceleration voltage of 80 kV, filament voltage preset at 19V, emission current preset at 10 mA, live image acquisition of 100 ms, capture exposure of 1600 ms and camera gain of 80. To visualize ascorbate peroxidase (APEX)-labeled mitochondria [14, 15], PASMCs were overlaid with H_2O_2 and 3, 3'-diaminobenzidine (DAB) to allow APEX-catalyzed polymerization for 25 minutes before staining with OsO4.

Measurement of H_2O_2 generation from mitochondrial suspension

The production of H_2O_2 from isolated mitochondria was measured using the method of Quinlan [16]. The cultured PASMCs were incubated with 2.25 10^8/ml GFP-labeled mitochondria prepared from FASMCs. 24 hours later, mitochondria were isolated from PASMCs or pulmonary arteries and subjected to flow cytometry for separation of endogenous mitochondria and exogenous, GFP-labeled mitochondria. The recovered mitochondria (~ 0.4 µg /ml mito protein) were suspended in saline of 120 KCl, 5 K_2PO_4, 2.5 $MgCl_2$, 1 EGTA, 5 HEPES (mM, pH 7.0 at 37°C) and 0.3% (wt/vol) bovine serum albumin, supplemented by 5 U/ml horseradish peroxidase, 25 U/ml SOD, and 50 µM Amplex UltraRed. Amplex UltraRed fluorescence of mitochondria was monitored before and after hypoxia on a BioTek Synergy2 microplate reader (Biotek Instruments, Winooski, VT) using a wavelength of excitation at 560 nm and emission at 590 nm, respectively. Hypoxia was achieved by bubbling the mitochondrial suspension with N_2 through a sealed tubing connected to the spectrophotometer. The background fluorescence was obtained in mitochondria suspended in the above saline without Amplex UltraRed. The changes of Amplex UltraRed fluorescence were corrected by subtracting the background and normalized by the quantity of mitochondrial protein.

Mitochondrial labeling with APEX for ultra-structural visualization

To label mitochondria for ultra-structural visualization, we adopted engineered ascorbate peroxidase (APEX), the genetic label for electron microscopy (EM) and a novel technique recently developed for ultra-structural visualization of mitochondria [14, 15]. The mitochondria targeted APEX-expressing vector was constructed following the strategy of pcDNA3-mito-APEX from Addgene (Cambridge MA). A mitochondria-targeting sequence of MLATRVFSLVGKRAISTSVCVRAH was inserted into the pcDNA3.0 vector (Invitrogen, V795-20) between NotI and BamHI to generate the pcDNA3-mito vector. The 870 bp full length cDNA of APEX (soybean, NM_016996) was synthesized artificially by Jurassic DNA Synthesizer (Invitrogen). The APEX cDNA was subcloned into the pcDNA3-mito vector with V5 (GKPIPNPLLGLDST) to fuse with the mitochondria-targeting sequence to obtain the pcDNA3-mito-APEX. The successful subcloning was verified by sequencing in forward and reverse directions by ABI3730 Automated Sequencer (Applied Biosystems) in more than two separate clones and the subsequent analysis with Seqman software from DNAstar package (Lasergene, USA). The pcDNA3-mito-APEX vector was transfected into FASMCs to label mitochondria and the APEX-labeled mitochondria were then isolated and prepared for the subsequent transplantation into PASMCs in culture. After a 24 hours of incubation with 2.25×10^8/ml APEX-labeled mitochondria, PASMCs were fixed with 2% glutaraldehyde in PBS and then overlaid with 3, 3'-diaminobenzidine tetrahydrochloride (DAB, 0.5 mg/mL) and H_2O_2 (0.03%, v/v) solution to allow APEX-catalyzed polymerization for 25 minutes. The DAB polymers were subsequently stained with osmium tetroxide (OsO4) before the final examination with a transmission electron microscope (FEI Tecnai G2 20 TWIN) together with an Olympus CCD (Cantega G2) and software (Cantega G2). APEX protein was obtained from Sigma.

Assay of SDH mRNA expression

Total RNA was extracted from PASMCs and FASMCs and subject to real-time PCR as described previously [17]. In brief, 1 mg total RNA was used to generate cDNA using Superscrip First Strand System (Takara). RNA and primer were gently mixed with 20 ml cDNA Synthesis Mix (Takara) and incubated at 37 °C for 15 min. The reactions were terminated by incubation at 85 °C for 5 min and then chilled on ice. Real-time PCR was performed with Sybgree1 Mix (Takara) on ABI Prism 7000 (Applied Biosystems). The specific primers for SDHα and β-actin were synthesized by Invitrogen with the sequences of 5'- AAC ACT GGA GGA AGC ACA CC-3' (forward), 5'-GCA ACT CGA GTC CCT CAC AT-3' (reverse) for SDHα, and 5'-ACG TTG ACA TCC GTA AAG ACC-3' (forward), 5'-AGG ATA GAG CCA CCA ATC CA-3' (reverse) for β-actin. The thermal cycle conditions were 30 s at 95°C and a 40-cycle loop: 5 s at 95°C, 20 s at 55°C and 30 s at 72°C. Duplicate measurements were conducted and the SDHα mRNA level was normalized by β-actin using 2^{-DDCT} method.

Measurement of cellular ATP content

The intracellular ATP levels were measured using an ATP Colorimetric/Fluorometric Assay Kit (Sigma) according to the manufacturer's instructions. Briefly, 1×10^6 cells were lyased in 100 µl ATP assay buffer. Then the cells were centrifuged at 12, 000 rpm for 5 min at 4°C, and the supernatant was collected. 50 µl supernatant was mixed with 50 µl the Reaction Mix to a well of the 96-well plate. The plate was incubated at room temperature for 30 min and then absorbance at 560 nm was measured using the microplate reader (TECAN, Sunrise). Standard curves were also generated using a series of standard ATP concentrations to calibrate the intracellular ATP levels. Triplicate measurements were performed for each sample.

Statistical analysis

Results were presented as Mean ± SEM. The analysis of Student's *t*-test or 1-Way ANOVA was performed for comparisons between 2 or $\geq$ 3 groups, respectively. Probability values < 0.05 were considered significant.

REFERENCES

1. Chen, T. *et al*. Mitochondria depletion abolishes agonist-induced Ca^{2+} plateau in airway smooth muscle cells: potential role of H_2O_2. *Am. J. Physiol. Lung. Cell. Mol. Physiol.* 298, L178-188 (2010).
2. Zhang, J. *et al.* Extracellular calcium-sensing receptor is critical in hypoxic pulmonary vasoconstriction. *Antioxid. Redox. Signal.* 17, 471-484 (2012).
3. King, M.P. & Attardi, G. Injection of mitochondria into human cells leads to a rapid replacement of the endogenous mitochondrial DNA. *Cell.* 52, 811-819 (1988).
4. Fernández-Vizarra, E., López-Pérez, M.J. & Enriquez, J.A. Isolation of biogenetically competent mitochondria from mammalian tissues and cultured cells. *Methods*. 26, 292-7 (2002).

5. Fernández-Vizarra, E. *et al*. Isolation of mitochondria for biogenetical studies: An update. *Mitochondrion*. 10, 253-62 (2010).
6. Roy, S. *et al*. Hyperoxia alters phorbol ester-induced phospholipase D activation in bovine lung microvascular endothelial cells. *Antioxid. Redox. Signal.* 5, 217-228 (2003).
7. Wang, Q.S. *et al*. Role of mitochondrial reactive oxygen species in hypoxia-dependent increase in intracellular calcium in pulmonary artery myocytes. *Free. Radic. Biol. Med*. 42, 642-653 (2007).
8. Westerblad, H. & Allen, D.G. The influence of intracellular pH on contraction, relaxation and $[Ca^{2+}]_i$ in intact single fibres from mouse muscle. *J. Physiol.* 466, 611-628 (1993).
9. Andrade, F.H., Reid, M.B., Allen, D.G. & Westerblad, H. Effect of hydrogen peroxide and dithiothreitol on contractile function of single skeletal muscle fibres from the mouse. *J. Physiol*. 509, 565-575 (1998).
10. Dooley, C.T. *et al*. Imaging dynamic redox changes in mammalian cells with greenfluorescent protein indicators. *J. Biol. Chem.* 279, 22284-22293 (2004).
11. Karunasinghe, R.N. & Lipski, J. Oxygen and glucose deprivation (OGD)-induced spreading depression in the Substantia Nigra. *Brain. Res.* 1527, 209-221 (2013).
12. Jin, H. *et al*. The role of sulfur dioxide in the regulation of mitochondrion-related cardiomyocyte apoptosis in rats with isopropylarterenol-induced myocardial injury. *Int. J. Mol. Sci.* 14, 10465-10482 (2013).
13. Zischka, H. *et al*. Liver mitochondrial membrane crosslinking and destruction in a rat model of Wilson disease. *J. Clin. Invest.* 121, 1508-1518 (2011).
14. Rhee, H.W. *et al*. Proteomic mapping of mitochondria in living cells via spatially restricted enzymatic tagging. *Science* 339, 1328-1331 (2013).
15. Martell, J.D. *et al*. Engineered ascorbate peroxidase as a genetically encoded reporter for electron microscopy. *Nat. Biotechnol.* 30, 1143-1148 (2012).
16. Quinlan, C.L., Treberg, J.R., Perevoshchikova, I.V., Orr, A.L. & Brand, M.D. Native rates of superoxide production from multiple sites in isolated mitochondria measured using endogenous reporters. *Free. Radic. Biol. Med*. 53, 1807-1817 (2012).
17. Song, S., *et al*. Irregular Ca^{2+} oscillations regulate transcription via cumulated spike duration and spike amplitude. *J Biol Chem*. 287, 40246-40255 (2012).

SUPPLEMENTARY FIGURES

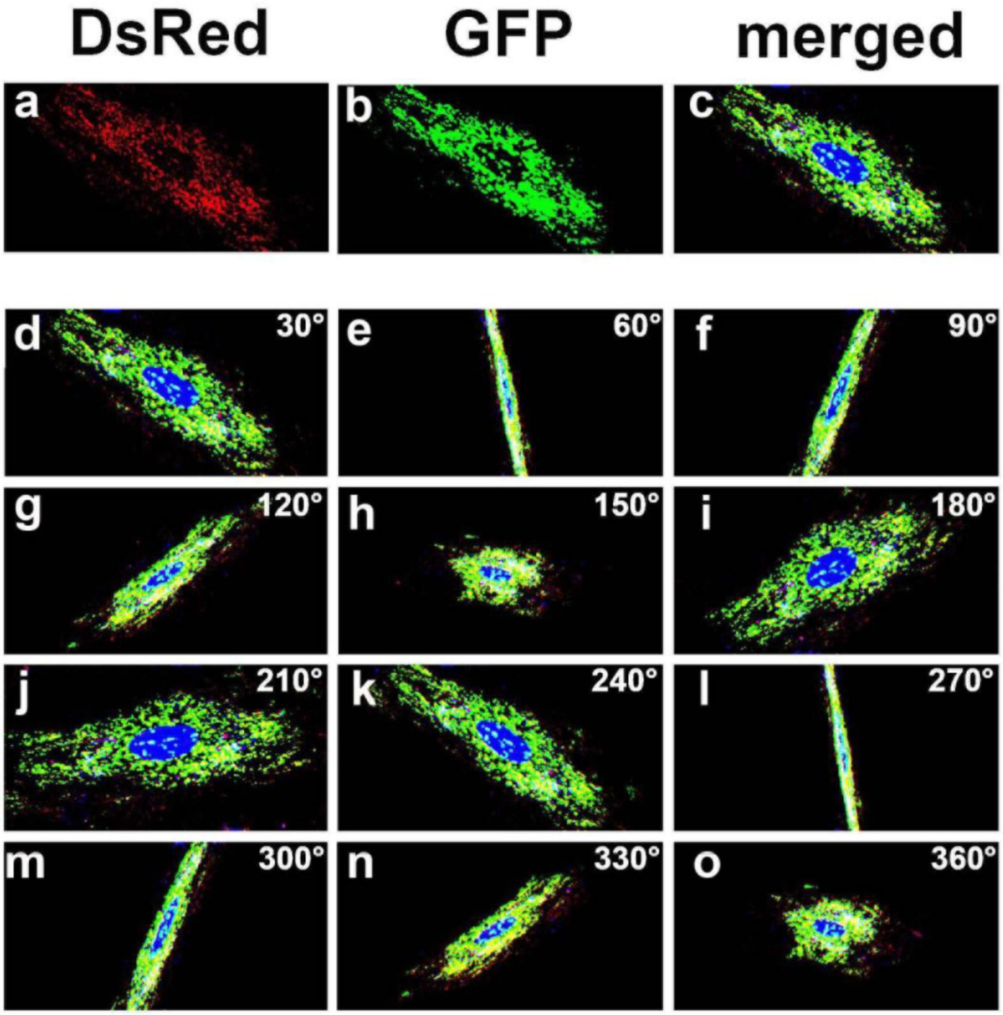

Supplementary Figure S1: Spatial distributions of transplanted mitochondria in PASMC. The three-dimensional resembling of continuous confocal scannings of every 0.3 μm from cell top surface revealing the spatial distributions of DsRed-labeled mitochondria (red) transplanted into PASMC with DiO- and DAPI-staining of cytoplasma membrane (green) and nucleus (blue), respectively. Shown were the sectional views of the three-dimensional picture continuously rotated in every 30° in X, Y and Z axis simultaneously (**c-o**, n=6).

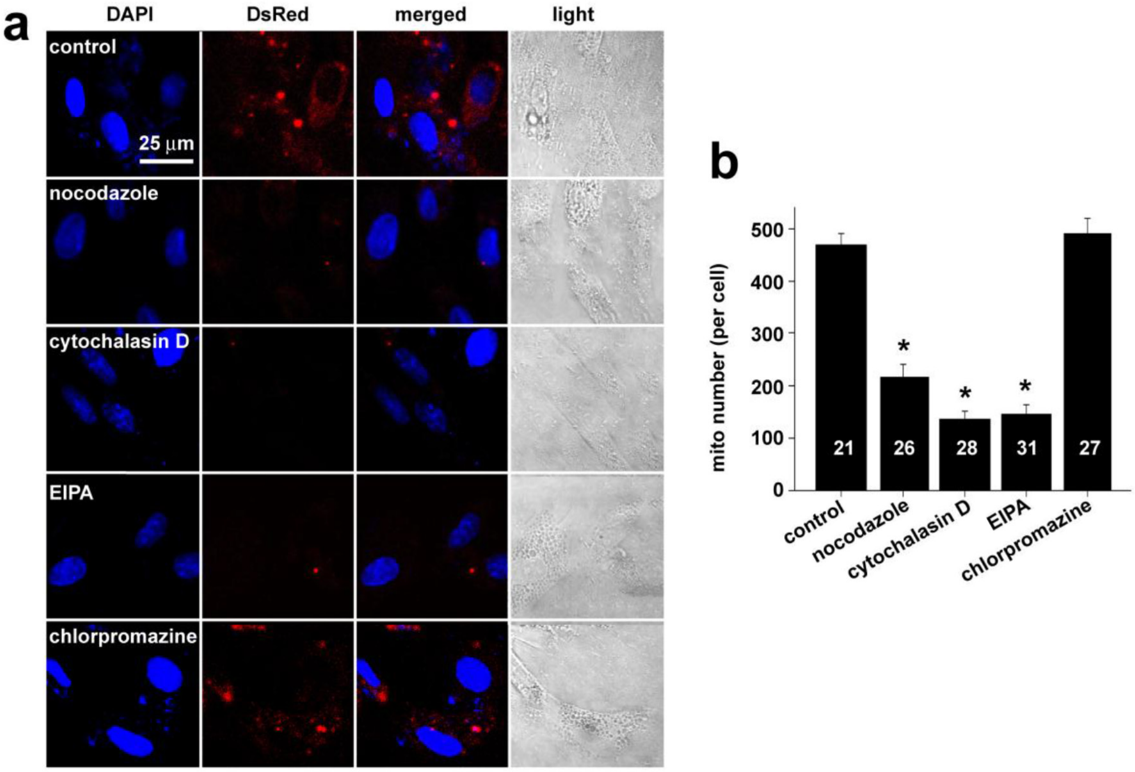

Supplementary Figure S2: Macropinocytosis-dependent internalization of exogenous mitochondria. The confocal images of pulmonary artery smooth muscle cells (PASMCs) after a 24 hours of incubation with DsRed-labeled mitochondria prepared from femoral artery smooth muscle cells in the absence or the presence of 10 μM nocodazole, 2 μM cytochalasin D, 25 μM 5- (N-ethyl-N-isopropyl) amirolide (EIPA) or 10 μM chlorpromazine, respectively **a.** and statistical summary (the number in the bar indicates individual PASMCs from 3-4 separate experiments, *$p < 0.05$ *vs* control, **b.**).

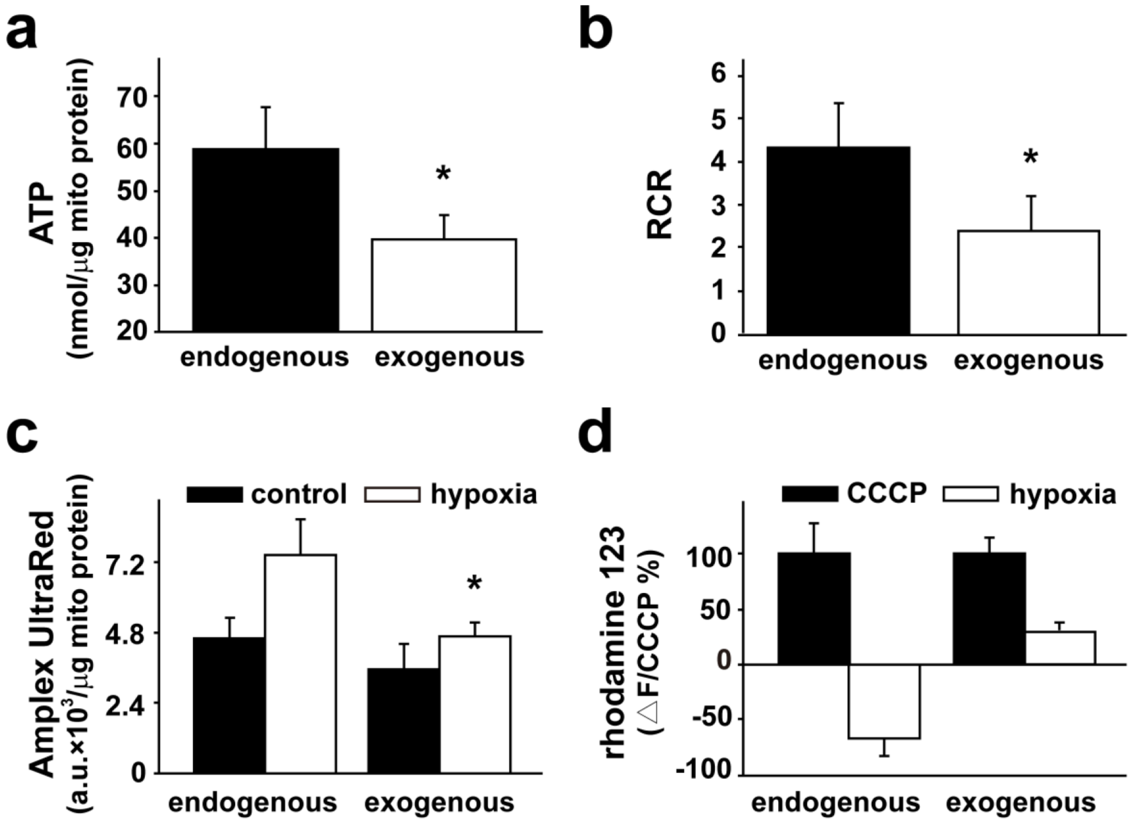

Supplementary Figure S3: Functional evaluation of endogenous and exogenous mitochondria recovered from pulmonary artery after transplantation of exogenous mitochondria prepared from femoral artery smooth muscle cells. Pulmonary arteries were incubated with 2.25×10^8/ml GFP-labeled mitochondria prepared from femoral artery smooth muscle cells at 4°C overnight. Mitochondria isolated from PAs were subjected to flow cytometry for the subsequent sorting and recovery of the endogenous, un-labeled mitochondria and the exogenous, GFP-labeled mitochondria. The recovered mitochondria were evaluated for their ability to produce ATP **a.**, respiratory control ratio (RCR, **b.**), their capability to generate ROS under hypoxia, respectively **c.** and their changes of mitochondrial membrane potential (MMP) in response to hypoxia **d.** $*p < 0.05$ vs. endogenous, n=3 for each (PAs from 3 separate rats).

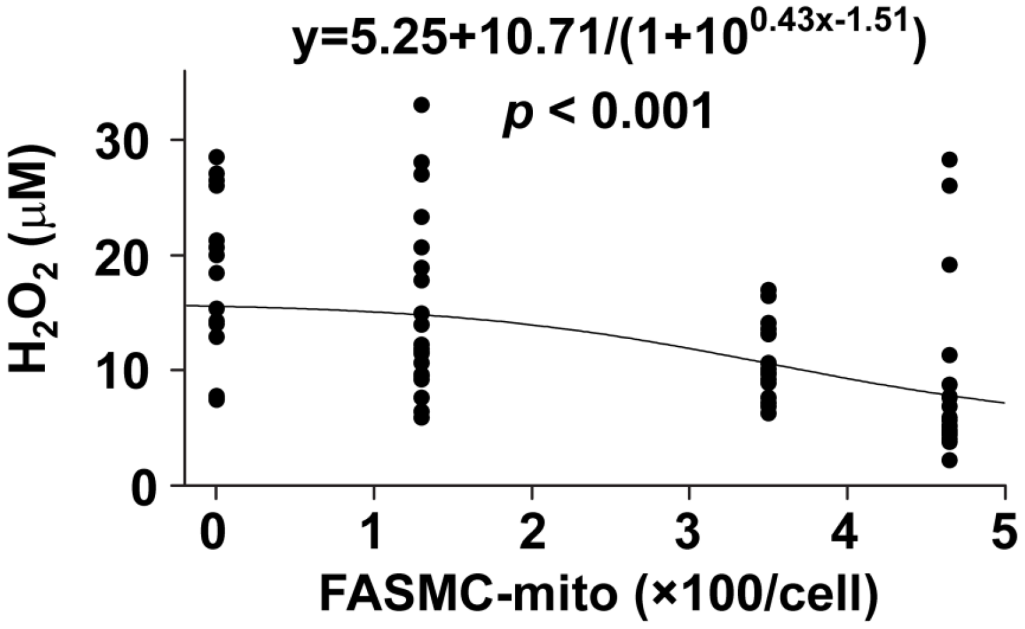

Supplementary Figure S4: The relationship between the number of intracellularly transplanted FASMC-mito and hypoxia-stimulated ROS levels in PASMCs. After incubation with 2.25×10^{8}/ml mitochondria prepared from femoral artery smooth muscle cells (FASMC-mito) for 6, 12 and 24 hours, which expectedly resulted in intracellular FASMC-mito at 130, 350 and 465 per PASMC, respectively, the PASMCs were loaded with DCFDA and evaluated for hypoxia-simulated ROS generation (n=19, 21, 18, 23 individual PASMCs for 0, 6, 12 and 24 hours, respectively, $p < 0.001$, four-parameter logistic regression).
